# High-Risk Non-Muscle-Invasive Bladder Cancer—Therapy Options During Intravesical BCG Shortage

**DOI:** 10.1007/s11934-016-0625-z

**Published:** 2016-08-04

**Authors:** Rajan Veeratterapillay, Rakesh Heer, Mark I. Johnson, Raj Persad, Christian Bach

**Affiliations:** 1 Department of Urology, Freeman Hospital, Newcastle upon Tyne Hospitals NHS Foundation Trust, Newcastle upon Tyne, NE77DN UK; 2 Bristol Urology Institute, Southmead Hospital, Bristol, UK

**Keywords:** Bladder cancer, Surgery, Bacilli Calmette-Guerin, Intravesical Therapy, Mitomycin C

## Abstract

Bladder cancer is the second commonest urinary tract malignancy with 70–80 % being non-muscle invasive (NMIBC) at diagnosis. Patients with high-risk NMIBC (T1/Tis, with high grade/G3, or CIS) represent a challenging group as they are at greater risk of recurrence and progression. Intravesical Bacilli Calmette-Guerin (BCG) is commonly used as first line therapy in this patient group but there is a current worldwide shortage. BCG has been shown to reduce recurrence in high-risk NMIBC and is more effective that other intravesical agents including mitomycin C, epirubicin, interferon-alpha and gemcitabine. Primary cystectomy offers a high change of cure in this cohort (80–90 %) and is a more radical treatment option which patients need to be counselled carefully about. Bladder thermotherapy and electromotive drug administration with mitomycin C are alternative therapies with promising short-term results although long-term follow-up data are lacking.

## Introduction

Bladder cancer is the second frequent urinary tract malignancy and out of all malignancies worldwide ranks as the 7th commonest male and 17th commonest female cancer [1]. The most common symptom at presentation is haematuria and diagnosis is usually made following transurethral resection (TUR). At diagnosis, 70–80 % of bladder cancer is non-muscle invasive (NMIBC) and of those 20–25 % progress to muscle-invasive disease during a patient’s lifetime [2]. Pathologically, NMIBC papillary tumours confined to the epithelium and those which have invaded the underlying lamina propria are classified as stage Ta and stage T1, respectively, while flat, high grade tumours that are confined to the mucosa are classified as carcinoma in situ (CIS/Tis) [3]. Following TUR and pathological analysis, NMIBC can be risk stratified into low, intermediate and high-risk groups depending on the probability of recurrence and progression to muscle-invasive disease [4]. Patients with high-risk NMIBC (T1, with high grade/G3, and/ or CIS) represent a challenging group with an increased 5-year risk of recurrence (up to 80 %) and progression (up to 50 %) according to the EORTC risk stratification tables [5].

Treatment options for high-risk NMIBC include intravesical immunotherapy with Bacillus Calmette-Guerin (BCG), other intravesical chemotherapy (with agents such as mitomycin C or epirubicin) or primary radical cystectomy. Intravesical BCG has been proven to reduce and delay tumour progression to muscle-invasive disease and has been first line therapy for many patients with high-risk NMIBC [6]. The response to intravesical treatment with BCG or chemotherapy is also an important prognostic factor for subsequent progression and death caused by bladder cancer [7–9]. Approximately 10–20 % of complete responders eventually progress to muscle-invasive disease, compared with 66 % of non-responders [10, 11]. There is a current worldwide shortage of intravesical BCG due to problems in manufacturing and limited supplies are available in centres [12]. Therefore, in this current climate, other treatment options for patients with high-risk NMIBC need to be considered.

This paper reviews the current evidence for intravesical BCG and alternative therapies for this challenging group of patients with high-risk non-muscle-invasive bladder cancer.

## Intravesical Immunotherapy with BCG

BCG is a live attenuated strain of *Mycobacterium bovis*, originally developed as a vaccine for tuberculosis. There are several strains available from different manufacturers with the commonest used in the UK being Oncotice BCG®. The content of a vial is reconstituted with 50 ml of saline and then administered through a urethral catheter to remain in the urinary bladder for about 2 h’ time. The mechanism of action is incompletely understood but it is postulated to work via a local immune response characterised by induced expression of cytokines in the urine and bladder wall [13]. Before starting BCG in new patients, it is important to adequately debulk all visible tumour and re-resect T1 high grade tumours to reduce understaging. BCG strain may have an impact on treatment outcome in NMIBC immunotherapy. A recent prospective randomised study of 142 high-risk NMIBC patients with two of the commonest BCG strains (Connaught and Tice) showed that treatment with BCG Connaught conferred significantly greater 5-year recurrence-free survival compared with treatment with BCG Tice [14].

Level 1 evidence from meta-analyses by Shelley et al. (2001) and Han et al. (2006) have confirmed the that BCG after TUR is superior to TUR alone or TUR and intravesical chemotherapy for the prevention of recurrence of NMIBC in patients with Ta and T1 tumours [15, 16]. Two other meta-analyses have demonstrated that BCG therapy prevents, or at least delays, the risk of tumour progression in patients at intermediate and high risk of tumour recurrence [17, 18]. As such, the landmark EORTC-GUCG analysis of over 4800 subjects from 24 RCTs comparing BCG (with maintenance) to other therapies (TUR alone, TUR with intravesical chemotherapy and TURB with other immunotherapy) showed a reduction of 27 % in the odds of progression with BCG maintenance treatment [18]. Particularly in the high-risk NMIBC group, studies have confirmed the superiority of BCG for prevention of tumour recurrence over intravesical mitomycin C alone, intravesical epirubicin alone or a combination of epirubicin and interferon [18–20]. These data therefore suggest that in patients with NMIBC at high risk of recurrence, BCG with at least 1 year of maintenance is more effective than chemotherapy for prevention of recurrence. Although BCG is a very effective treatment, there is a consensus that not all patients with NMIBC should be treated with BCG due to the risk of toxicity [21, 22]. Whilst the induction BCG instillations are classically given according to the empirical 6-weekly induction schedule that was introduced by Morales, the BCG maintenance schedule has not been standardised [23]. One common maintenance BCG schedule used in clinical trials would consist of three weekly instillations at months 3, 6 and 12 following induction treatment [6, 17].

With limited supplies of intravesical BCG available, judicious use of the dwindling reserves must be made. For patient already on treatment, BCG maintenance can be stopped after 1 year in patients without CIS and continued for up to 3 years in patients with CIS at a reduced (1/3) dose (Table 1, option 1). This schedule does not seem to lead to more tumour progression, although a higher risk of recurrence has been reported in the patients with very high risk [31].

**Table 1:** Options in BCG shortage

Option 1 [19]	Full dose BCG with maintenance only for 1 year (if CIS continue for 2–3 years)
Option 2 [19]	One third dose BCG for induction and maintenance for 1 year
Option 3 [6, 21]	Induction and maintenance course of mitomycin C
Option 4 [24, 25]	Intravesical chemotherapy agents with thermotherapy
Option 5 [26, 27]	Induction and maintenance course of gemcitabine
Option 6 [28–30]	Primary radical cystectomy

Another option for new patients would be using a reduced dose of 1/3 BCG for induction and maintenance for 1 year (Table 1, option 2). This schedule has been investigated by the an EORTC-GU trial and could not show a difference in the number of patients progressing as compared to full dose rate [31]. Alternatively, some expert recommendation suggest full dose BCG to be given as a 6-week induction course followed by maintenance at 1/3 dose [12]. However, if there is no or limited BCG available, primary cystectomy should be offered to all surgically fit patients with high-risk NMIBC as the most effective option compared with other non BCG-based treatments. Should patients have a recurrence of their high-risk NMIBC under BCG treatment, expert judgement advises no further challenge with BCG should be offered but radical cystectomy or device-assisted therapy should be considered [12].

## Intravesical Mitomycin

Mitomycin C with maintenance has been shown to reduce the risk of recurrence in intermediate and high-risk NMIBC, but it is not as effective as BCG. Trials of intravesical mitomycin C versus BCG can be difficult to interpret due to heterogeneous treatment groups and variable intervention schedules. Three main RCTs have dealt specifically with patients at high risk of recurrence [32–34]. Those include patients with recurrent Ta/T1 tumours, T1G3 tumours or concomitant CIS.

Rintala et al. reported an RCT of 109 patients with recurrent Ta/T1G1-3 tumours or CIS treated (using weekly installation of BCG for 4 weeks followed by two monthly instillations up to 1 year) and found a complete response to BCG (defined as lack of recurrence) in 97 % (vs 70 % for maintenance mitomycin using same similar schedule) at 2-year follow-up [33]. In another RCT of 469 patients from the SWOG group (patients at high risk due to having had two occurrences of tumour within 56 weeks, stage T1 tumour or three or more tumours within 16 weeks, or concurrent CIS), recurrence-free survival was significantly longer with BCG but there was no difference in progression [34]. In another study by Malstorm et al., 126 patients with multiple or recurrent Ta/T1G1-2 or CIS or T1G3 were randomised to BCG with maintenance or mitomycin C [24]. The authors reported a significant difference in disease-free rates at 5 years (47 % for BCG arm vs 34 % for mitomycin) but no difference in progression rates.

In an individual patient data meta-analysis (over 2800 patients) of nine randomised studies comparing long-term outcomes of intravesical therapies in NMIBC, the trials with BCG maintenance showed a 32 % reduction in risk of recurrence on BCG compared to mitomycin while there was a 28 % risk increase for BCG in the trials without maintenance. There was however no statistically significant differences regarding progression, overall survival and cancer-specific survival between the two treatments [25]. These data show that intravesical BCG with maintenance has superior activity compared to mitomycin C (MMC) for the prophylaxis of tumour recurrence in high-risk patients. However, it is worth noting that in the RCTs, there was a response rate associated with use of maintenance mitomycin C as regards the reduction in recurrence rates even in high-risk patients. For this reason whilst accepting a higher risk of recurrence, an induction course of MMC followed by maintenance seems to be a viable alternative to BCG in times of shortage, especially for high-risk NMIBC patients without features putting them at the highest risk (pT1G3 with CIS for example) (Table 1, option 3). Due to the current lack of evidence as to which schedule is the most efficient, we currently deliver a 6-week induction course followed by weekly maintenance for 1 year with cystoscopic assessment at 3 months.

## Intravesical Chemotherapy with Hyperthermia

Thermal energy appears to improve the absorption of several intravesical agents, and as such adjunctive hyperthermia has been shown to increase cytotoxicity in bladder cells in in vitro and animal models [35–37]. Several devices are available to deliver thermochemotherapy for NMIBC with the commonest used heat delivery system being Synergo® (Medical Enterprises Europe B.V., Amsterdam, Netherlands). This machine delivers local hyperthermia in conjunction with intravesical chemotherapy using a 915-MHz intravesical radiofrequency energy applicator placed on a thermocouple-controlled 20 F urethral catheter [12]. Other HT systems include the BSD-2000 (currently used for high-risk bladder cancer part of a quadrimodal treatment plan involving TUR, radiation therapy, chemotherapy and hyperthermia), the ALBA hyperthermia system (which includes a 3D system), the BWT system by Elmedical, the Sonotherm 1000 and the Combat BRS system [38–40].

A recent systemic review and meta-analysis of 22 trials examining intravesical chemohyperthermia (CHT) for NMIBC showed a 59 % reduction in recurrence rate for TURB + CHT compared to TURBT + chemotherapy alone [41]. However, due to short follow-up, no conclusions could be drawn about time to recurrence and progression. In addition, the overall bladder preservation rate after CHT was 87.6 %.

In one of the few studies with long-term follow-up, Colombo et al. reported on a cohort of 83 patients with intermediate/high grade NMIBC (randomised to undergoing thermotherapy with Synergo vs standard mitomycin C) with a median follow-up of 91 months [42]. They showed a significantly better 10-year disease-free survival rate for thermochemotherapy (53 vs 15 % for mitomycin only). Bladder preservation rates for thermochemotherapy were also significantly higher (86 vs 79 %).

The European Synergo working party recently reported outcomes of CHT with mitomycin C in CIS patients. In this multicentre study of 51 patients with primary or BCG-failing CIS, the authors reported an initial complete response rate of 92 %, which remained approximately 50 % after 2 years [43].

Overall, the early evidence suggests that chemohyperthermia offers an additional benefit to current clinical management methods, as an alternative treatment should BCG not be available, especially for higher risk NMIBC patients with T1 tumours or CIS for whom upfront radical cystectomy is not an option (Table 1, option 4). More studies are needed to further examine long-term oncological outcomes, treatment complications and limitations of this approach.

## Electromotive Intravesical Drug Administration

Electromotive drug administration (EMDA) uses an electric current to enhance transepithelial drug penetration [44]. EMDA is administered via a battery-powered generator delivering an electric current of 0–30 mA DC at 0–55 V, which is passed between two electrodes. An active electrode is placed into the bladder as part of a transurethral catheter and dispersive ground electrode pads are placed on the skin of the lower abdomen [26].

The penetration of MMC into the bladder wall with EMDA is significantly greater (by four to seven times) than that achieved by passive diffusion as shown in in vitro studies [27, 45]. In clinical practice, EMDA MMC has been shown to be safe with no life-threatening adverse events. Most reports on EMDA have focussed on its role following first TURBT and there are limited reports of its use in high-risk patients.

In one such study, Di Stasi et al. reported a prospective RCT of 108 patients (with multifocal Tis, including 98 with T1 tumours) who were randomised into three equal groups of 36 each who underwent 40 mg electromotive MMC instillation with 20 mA electric current for 30 min, 40 mg passive MMC with a dwell time of 60 min or 81 mg BCG with a dwell time of 120 min. The complete response for electromotive versus passive MMC at 3 and 6 months was 53 versus 28 % (*p* = 0.036) and 58 versus 31 % (*p* = 0.012). For BCG, the responses were 56 and 64 %. Median time to recurrence was 35 versus 19.5 months (*p* = 0.013) and for BCG it was 26 months [28].

In another RCT of 212 patients comparing sequential BCG and electromotive mitomycin C versus BCG alone for high-risk superficial bladder cancer, patients assigned sequential BCG and electromotive mitomycin had higher disease-free interval than did those assigned BCG alone (69 vs 21 months) [29]. Patients assigned sequential BCG and electromotive mitomycin also had lower recurrence (41.9 vs 57.9 %), progression (9.3 vs 21.9 %), overall mortality (21.5 vs 32.4 %) and disease-specific mortality (5.6 vs 16.2 %).

These data suggest that intravesical electromotive administration appears to increase bladder uptake of MMC, resulting in an improved response rate in cases of high-risk NMIBC. This appears equivalent to BCG in the short term but long-term efficacy data are lacking. Therefore, it is currently not possible to give a conclusive recommendation about the place for this modality in times of BCG shortage.

## Other Intravesical Agents

### Doxorubicin

Doxorubicin is an anthracycline used in superficial bladder cancer. RCTs comparing it to BCG have shown that BCG is superior in preventing or delaying tumour recurrence both in papillary Ta and T1 cancers and CIS [30, 46, 47].

### Intravesical Gemcitabine

A Cochrane review on the efficacy of gemcitabine showed six trials with over 700 patients [48]. Three trials compared gemcitabine with intravesical BCG but a meta-analysis was not possible due to clinical heterogeneity. In untreated patients at intermediate risk of recurrence (primary Ta-T1 no CIS), one trial showed that gemcitabine and BCG were similar with respective recurrence rates of 25 and 30 % and overall progression equal. In a second trial of high-risk patients, the recurrence rate was significantly greater with gemcitabine compared to BCG (53.1 and 28.1 %) and the time to recurrence significantly shorter with gemcitabine (25.5 vs 39.4 months). Finally in a third trial of high-risk patients who had failed previous intravesical BCG therapy, gemcitabine was associated with significantly fewer recurrences (52.5 vs 87.5 %) and a longer time to recurrence (3.9 vs 3.1 months) compared to BCG. Progression rates were similar in both groups (33 vs 37.5 %) with no significant differences in grade 2 or 3 toxicities. In summary, compared to intravesical BCG therapy, gemcitabine had similar effects in intermediate risk patients but was less effective in high-risk patient and remains frequently reserved for BCG refractory patients. A proposed schedule for example is induction with intravesical gemcitabine (1–2 g in 50 cc of sterile water over 90 min) followed by a monthly maintenance (Table 1, option 5) [49].

## Radical Cystectomy

Radical cystectomy has been considered the gold standard treatment for patients with high-risk NMIBC, such as multiple and/or large T1G3 with CIS or recurrent high-risk NMIBC after intravesical full dose BCG for 1–3 years [21, 50].

The high risk of recurrence and progression associated with conservative treatment of high-risk NMIBC indicates there is a considerable risk of understaging by TUR and imaging alone [51]. Cystectomy allows pelvic lymphadenectomy which permits accurate staging (up to 18 % of T1 patients have positive lymph nodes) and can be therapeutic regarding nodal metastases [52]. Herr and Sogani reported on 307 high-risk bladder cancer patients of whom 90 underwent cystectomy for relapse. Cancer-specific survival (CSS) was greatest in those undergoing cystectomy <2 years after BCG initiation compared to those whose cystectomy was performed >2 years after BCG induction [53]. Lambert et al. also noted poorer recurrence-free survival (RFS) and CSS in a cohort of patients undergoing cystectomy for T1G3 carcinoma after 1998 versus those treated with cystectomy before 1998. They postulated that the decrease in survival was the result of higher and perhaps inappropriately prolonged use of intravesical therapy in the modern era [54]. The above data highlights the danger of delaying cystectomy in patients with highest-risk NMIBC and the need to risk-stratify all such T1 patients. The ability to identify candidates at high risk for progression is indispensable in selecting patients who would benefit from primary or early cystectomy.

However, if no BCG is available, patients who would not be classified as the highest risk category may still be considered for upfront radical cystectomy as the safest option from the oncological point of view (Table 1, option 6). Long-term oncological outcomes of cystectomy for those patients show disease-specific survival of 80–90 % [51].

## Conclusions

High-risk NMIBC represents a challenging cohort of patients where the risk of recurrence and progression has to be balanced against treatment morbidity. Intravesical BCG has been first line treatment for many of those patients but there is a current worldwide shortage.

In this climate, judicious use of limited BCG supply should be made. Therapeutic choices in this patient group are listed in Table 1. For patients in the high-risk group without CIS, maintenance BCG can be stopped after 1 year. For those with CIS, a reduced dose of BCG for years 2 and 3 could be considered.

An alternative strategy for patients at lower risk of progression (pTaG3) would include an induction course of mitomycin C that should be followed by maintenance as level 1 evidence suggests a response rate even in the high-risk group associated with MMC.

Thermotherapy or EMDA with mitomycin have both shown good response rates but the studies have been limited with short follow-up data. Recruitment of patients in clinical trials would be helpful.

In patients fit for surgical intervention, as strong case can be made for radical cystectomy and patients need to be counselled about this prior to embarking upon intravesical therapies.
